# Virtual Mentored Implementation to Improve Care Transitions in Chronic Obstructive Pulmonary Disease: Protocol for a Pragmatic Implementation Study

**DOI:** 10.2196/82043

**Published:** 2026-01-13

**Authors:** Cathryn T Lee, Leah Traeger, Mahima Akula, April E Fegley, Jenna Goldstein, Kim Erwin, Laura J Damschroder, Jean Rommes, Hannah Pick, Andrew Auerbach, Peter Lindenauer, Wen Wan, Andrea Jackson Sagredo, Valerie G Press

**Affiliations:** 1 Section of Pulmonary and Critical Care Medicine, Department of Medicine University of Chicago Chicago, IL United States; 2 Society of Hospital Medicine Philadelphia, PA United States; 3 Institute of Design Illinois Institute of Technology Chicago, IL United States; 4 Implementation Pathways LLC Chelsea, MI United States; 5 Chronic Obstructive Pulmonary Disease Foundation Des Moines, IA United States; 6 Onda Collective LLC Chicago, IL United States; 7 Division of Hospital Medicine University of California, San Francisco San Francisco, CA United States; 8 Division of Healthcare Delivery and Population Sciences UMass Chan Medical School- Baystate Springfield, MA United States

**Keywords:** COPD, chronic obstructive pulmonary disease, hospital readmissions, transitions of care, COPD exacerbation, hybrid effectiveness-implementation design

## Abstract

**Background:**

Chronic obstructive pulmonary disease (COPD) is a leading cause of mortality and morbidity among US adults, including recurrent emergency department (ED) visits and unplanned hospital admissions. Despite this, the transition of care (TOC) from the inpatient to outpatient setting remains under-studied.

**Objective:**

The objectives of the Reduce Respiratory Emergent Visits using Implementation Science Interventions Tailored to Setting (Reduce REVISITS) study are to conduct contextual assessments to inform implementation plans for COPD TOC interventions, conduct a cluster randomized trial evaluating implementation over 1 year of COPD TOC bundles, and monitor sustainment of implementation over a 2-year period across 20 sites.

**Methods:**

This pragmatic, multisite study uses a hybrid type II effectiveness-implementation design to evaluate clinical and implementation outcomes of COPD TOC programs across 20 sites. Sites are cluster-randomized to 1 of 4 intervention groups, varying by program delivery method (in-person vs virtual) and implementation strategy (mentored implementation with or without co-design). Sites select evidence-based interventions they wish to incorporate into their COPD TOC program and are randomized to in-person or virtual delivery. During the 1-year active implementation period of the study, assigned mentors will meet monthly with sites (for a total of 12 sessions) to enable on-the-ground troubleshooting of site-specific difficulties with TOC interventions. The primary effectiveness outcome for this study will be COPD-specific acute health care use, defined as a composite of all ED visits and hospitalizations within 30 days of index hospitalization for a COPD exacerbation. The primary implementation outcome will be reach, defined as the proportion of patients receiving their assigned TOC interventions (the whole bundle).

**Results:**

As of August 2025, 21 sites completed the contextual assessments and developed site-specific implementation plans. Publication of the qualitative data from this pre-implementation phase is anticipated in December 2025. Site randomization is complete; sites randomized to co-design have completed 3 sessions. Baseline data collection on use is complete. Implementation-year data collection on use is nearly complete. Year 1 and 2 post-implementation-phase data collection on use is ongoing. Quantitative data analyses of the baseline and implementation-phase reports are nearly complete. Manuscript submission for the primary implementation-phase manuscript is anticipated for December 2025. Manuscript submission for the implementation-sustainment analyses are anticipated for September 2026. Qualitative data collection for year 1 of the post-implementation phase is complete, and analysis is under way. Qualitative data collection for year 2 began in August 2025.

**Conclusions:**

The Reduce REVISITS study will use novel integrated implementation science and human-centered design methodology to investigate bundles of effective COPD TOC interventions with the goal of reducing COPD hospital revisits. The study will evaluate evidence-based programs for effectiveness and implementation across a wide variety of health care sites to ultimately improve outcomes in this high-risk patient population.

**Trial Registration:**

ClinicalTrials.gov NCT05568043; https://clinicaltrials.gov/study/NCT05568043

**International Registered Report Identifier (IRRID):**

DERR1-10.2196/82043

## Introduction

Chronic obstructive pulmonary disease (COPD) is one of the leading causes of morbidity and mortality for US adults [1], but the effectiveness of implementing COPD chronic disease management programs has been under-studied. This dearth of data became salient in 2014 when the Centers for Medicare and Medicaid Services added COPD to their Hospital Readmission Reduction Program (HRRP), which financially penalizes hospitals receiving federal funding for excessive 30-day readmissions [2]. Since COPD was the third leading cause of readmission among Medicare beneficiaries [3], including COPD among penalized conditions was logical. However, an expert panel convened in 2016 by the American Thoracic Society (ATS) to identify the best evidence and practices to reduce readmissions for acute exacerbations of COPD found that at the time of HRRP implementation there was little published evidence on effective hospital-based programs to reduce readmissions [4].

In the decade since the HRRP implementation, a handful of interventions have shown promise at reducing readmissions and facilitating transitions of care (TOC) from the inpatient to outpatient setting. These include early pulmonary rehabilitation, inhaler education, medication reconciliation and education via pharmacists, smoking cessation counseling, and early outpatient follow-up. However, a single intervention is unlikely to reduce readmissions enough to avoid financial penalties [5]. Hence, bundles of interventions through transition of care programs hold more promise. One study evaluated a COPD Chronic Care Management Collaborative comprised of education through subject matter experts and implementation support through peer coaching [6]. This collaborative aimed to support sites’ implementation of interventions through quality improvement initiatives to reduce emergency department (ED) and hospital revisits across 47 US hospitals. With only about half of sites reporting, the collaborative found that they were able to successfully support the majority of sites in reducing ED and hospital revisits. In addition to this collaborative, a handful of institutions have published their attempts to address preventable acute care revisits after COPD hospitalization with mixed results [7]. For instance, one single-site randomized controlled trial (RCT) evaluating a 3-month transitional care and long-term self-management program for patients hospitalized with COPD found that patients enrolled in the intervention unexpectedly experienced harm in the form of more COPD acute care visits without improvement in quality of life [8]. Single-center studies of COPD bundled programs responding to the voluntary Bundled Payments for Care Innovation value-based care initiative of the Centers for Medicare and Medicaid Services have demonstrated mixed results regarding the effectiveness of readmission rates as well as the impact on overall health care costs [9,10].

One of the key concerns from the ATS report was a dearth of rigorously obtained data using RCTs along with limited sample size and consequently statistical power in single-center studies. Thus, we sought to study multiple COPD TOC bundle of care programs simultaneously in the National Institutes of Health–funded virtual mentored implementation to reduce Respiratory Emergent Visits using Implementation Science Interventions Tailored to Setting (Reduce REVISITS) study (R01HL146644). The purpose of this study is to evaluate tailored COPD TOC program delivery (in-person versus virtual) with implementation support (mentoring with or without co-design support) to identify ideal, scalable solutions for reducing excessive preventable COPD acute care revisits across diverse health care institutions. We used implementation science and human-centered design methods to develop the site-specific implementation plans [11]. We also will use these innovative methods to conduct the trial, specifically a hybrid type II effectiveness-implementation cluster RCT as a novel approach for rigorously and efficiently improving standards of care for TOC in high-risk patients with COPD. This hybrid approach will allow us to simultaneously evaluate the method of delivering the evidence-based interventions (in person versus virtual) along with measuring the level of implementation support (mentoring with or without co-design). The objectives of the Reduce REVISITS study are to (1) conduct contextual assessments using mixed methods to inform site-specific implementation plans for 2-3 evidence-based COPD TOC interventions (aim 1, methods published separately) [11]; (2) conduct a cluster randomized trial evaluating implementation over one year of the COPD TOC bundles (aim 2); and (3) monitor sustainment of implementation over a 2-year period (aim 3).

## Methods

### Study Design

**Figure 1:**
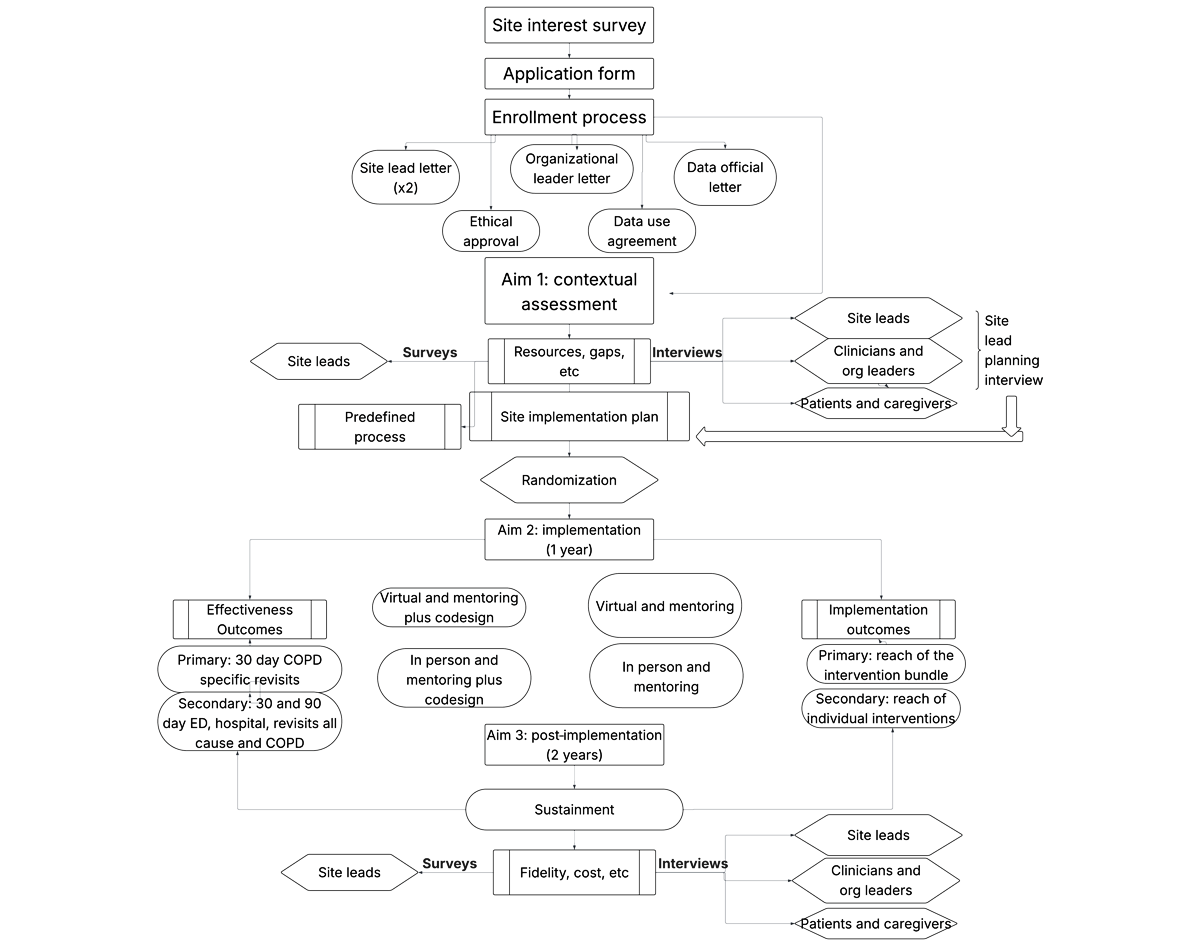
Overall study schematic. This protocol paper focuses on the aim 2 methods. COPD: chronic obstructive pulmonary disease; ED: emergency department.

This pragmatic, multisite study uses a hybrid type II effectiveness-implementation design to evaluate both clinical and implementation outcomes of COPD care transition programs across 20 diverse US hospital sites. After recruitment and enrollment, sites underwent a contextual assessment to develop site-specific implementation plans (published elsewhere) in aim 1 [11]. Participating hospitals will then be cluster-randomized to implement their intervention bundles using their site-specific implementation plans. Sites will be randomized to one of 4 intervention implementation groups, varying by program delivery method (in-person vs virtual) and implementation strategy (mentored implementation with or without co-design). Each site implements a tailored bundle of 2-3 evidence-based care transition interventions guided by the Society of Hospital Medicine (SHM)’s mentored implementation model (MIM) over a one-year period. Half of the sites also receive co-design support to actively engage end-users in shaping implementation approaches. The intervention groups are assessed through both quantitative outcomes, including acute care use, such as 30-day COPD-related revisits (primary effectiveness outcome) and bundle reach (primary implementation outcome). Deidentified patient-level data will be provided via Research Electronic Data Capture (REDCap; Vanderbilt University) and analyzed as below [12]. After one year of implementation, the project will enter the sustainment phase (aim 3), where ongoing effectiveness data and implementation data will be collected for an additional 2 years. Further, sustainment, fidelity, and cost will be evaluated through surveys and interviews. These post-implementation outcomes are measured over an additional 2-year follow-up period to assess the long-term sustainment and impact of implementation strategies and to inform future scale-up and dissemination (Figure 1). This study has been registered on ClinicalTrials.gov (NCT05568043).

### Population and Setting

Hospitals interested in improving their COPD TOC programs for patients hospitalized with COPD will be recruited into the initial phase of the study. The sites will be primarily identified from the Hospital Medicine Reengineering Network (HOMERuN), a collaborative of hospitals and care teams aimed at creating and improving best practices via a learning network [13]. The HOMERuN sites are geographically diverse and include both academic and community hospitals. Additional sites will be recruited through the SHM [14]. The study aims to enroll at least 20 hospitals in the first phase (aim 1). To allow for sufficient power of the co-primary outcomes (see power calculation below) with up to 20% attrition in between this contextual assessment (aim 1) and randomization (aim 2), a minimum of 16 of the 20+ sites recruited into aim 1 sites are needed to participate in the trial (aims 2 and 3).

### Site Recruitment and Onboarding

Sites are selected into the first phase of the study via a 3-step process designed to ensure diversity, feasibility, and readiness [15]. First, a site interest survey is disseminated broadly across the HOMERuN and SHM networks and through word-of-mouth referrals to gauge willingness and capacity to participate. Second, interested sites complete a detailed application capturing information about their hospital type (academic vs community), location (urban, suburban, and rural), baseline COPD care transition practices, population demographics, institutional priorities, and motivation for participating. The application also included questions to identify potential site leads and assess internal resources needed to support COPD program implementation.

Third, the formal enrollment process requires each site to (1) identify and commit at least 2 site leads to serve as project leads and points of contact; (2) submit letters of support from senior hospital leadership demonstrating institutional commitment; (3) secure institutional review board or ethical oversight approval; (4) confirm ability to collect, deidentify, and securely transmit data on a monthly basis via REDCap; and (5) engage in pre-implementation planning activities, including site lead interviews, providing names and contact information for stakeholder interviews, and discussions to develop a tailored implementation plan. All survey responses, applications, and enrollment forms are submitted via REDCap and tracked by the study team.

Once onboarding is complete, each enrolled site will participate in aim 1 activities, including contextual assessments and development of their site-specific implementation plans for 2-3 evidence-based COPD TOC interventions. Sites become eligible for randomization into aim 2 only after completing all aim 1 activities.

### Mentor Recruitment, Onboarding, and Training

Mentors will be recruited using a snowball approach from membership in the SHM and ATS [16]. Mentors will be expected to have quality improvement expertise. An onboarding process will consist of the SHM team and Reduce REVISITS team meeting virtually with mentors. Content will include processes related to baseline data collection and data reporting.

### Evidence-Based Interventions for TOC

During phase 1, individual sites select evidence-based interventions they wish to incorporate into their COPD TOC program (Table 1) [11]. Sites will then be randomized to deliver these COPD TOC programs either in person or virtually. All selected interventions are either part of standard clinical practice or supported by strong evidence for effectiveness. These interventions include, first, COPD action plans; these are structured tools that patients and health care providers review together to support patient self-management. Both the American Lung Association and the COPD Foundation have published free action plans that were available for use by sites [17,18]. Second, the interventions include gold standard inhaler education delivered using the “teach-to-goal” method [1,19,20]. Teach-to-goal adheres to the guideline-recommended “teach-back” technique [21] and is an iterative, patient-centered approach that ensures correct inhaler technique through cycles of teaching and testing (“teach-back”). It adapts to diverse learning needs and delivery methods and has demonstrated lasting improvements in both inhaler technique and health outcomes, making it a best-practice method in COPD inhaler education [19,22]. Third, medication reconciliation enhancements were also available for sites to adopt or expand upon, aiming to improve adherence and patient safety [23]. Fourth, post-discharge follow-up visits were used, whether in-person or virtual, as they have been associated with lower 90-day readmission rates; sites could opt to initiate new visit types or strengthen existing workflows and show rates [24,25]. Similarly, sites could choose to focus on patient navigation programs using community health workers, which draws on successful asthma models to support care continuity and address social determinants of health [26]. Fifth, smoking cessation assistance is provided, as it has proven associations with reduced mortality, slowed disease progression, and decreased acute care use in COPD patients [27,28]. Sixth, pulmonary rehabilitation is included in the interventions, as it is a well-established intervention that lowers readmission risks [29]. Sites had the option to launch a new program or work to enhance referrals and attendance in existing programs. Seventh, improved diagnostic testing through increased spirometry testing was also offered as an intervention option, addressing frequent underdiagnosis or misdiagnosis of COPD [30]. Eighth, sites had the option to update electronic health record order sets for COPD patients to streamline inpatient and outpatient COPD management by standardizing best practices for diagnosis and treatment. Consults for COPD care, general COPD education, and standardized note templates incorporating the above best practices were also interventions that could be included in site plans.

**Table 1:** Data supporting implementation of selected interventions aimed at reducing readmissions.

Intervention	In-person outcomes or care standards	Virtual outcomes or care standards	References
Post-discharge follow-up visit	Reduction in 90-day readmissions	Noninferior to traditional home-based post-discharge program in time to first exacerbation	Saxena et al [31] Mínguez Clemente et al [32]
Inhaler teach-to-goal	Reduction in 30-day acute care visits	Noninferior in rates of inhaler misuse	Press et al [33] Press et al [34]
COPD action plan	Standard practice	Faster exacerbation recovery time compared to no action plan	Farias et al [35]
Smoking cessation education	Hospital-based education program increased prevalence of cessation at 12 months	High rates of quit attempts and satisfaction with services received	Fung et al [36] Chase et al [37]
Medication reconciliation	Toolkits available to disseminate best practices	Pilot study was feasible and resulted in a high number of discrepancies recognized	Mueller et al [38] Heyworth et al [39]
Pulmonary rehabilitation	Reduction in 30-day readmissions, increase in one-year readmissions	Reduction in 30-day readmissions	Myers et al [40] Bhatt et al [41]
Remote monitoring	No “in-person” option	Some reduction in COPD-related readmissions Low quality of evidence, no improvement in quality of life or mortality	Janjua et al [42] Nagase et al [43]

### Implementation Support via MIM With or Without Co-Design

The MIM is a well-validated strategy to enhance the implementation, effectiveness, and sustainability of hospital-based quality initiatives (Table 2). Mentors will be randomly assigned to either one or 2 sites, based on their indicated time commitment to the study. For mentors with 2 assigned sites, both sites will be in the same randomized group (MIM only or MIM plus co-design). Mentor training will be in the form of a 2-day virtual “mentor university,” during which time they will receive best practices training in mentoring, delineate their roles and responsibilities, and discuss “case studies” of anticipated implementation issues they may encounter.

During the active implementation period of the study, mentors will meet monthly with their assigned sites (over the course of one year, for a total of 12 sessions) to enable on-the-ground troubleshooting of site-specific difficulties with TOC interventions. Monthly mentor calls will be highly structured and consist of initial updates of current events that could impact hospitalization rates, review of data, overview of implementation progress, review of plan-do-study-act cycles, brief review of further action items, and finally sustainability planning.

In addition to monthly mentoring sessions, mentors will also perform a virtual site visit (within the first several months of TOC implementation), allowing them to observe and respond to challenges occurring at the site level. These visits will be completed during the active implementation period of the study and consist of several meetings with multiple stakeholder groups (including the core intervention team, executive leadership, and implementing care providers). Templates of agendas and presentations will be provided to the mentors for their site-specific revisions, and site visit debriefs will be developed by mentors at the conclusion of the site visit to document the team’s ongoing efforts, direct their focus toward opportunities for greater adherence, and potentially garner additional institutional support.

Half of the sites will additionally be randomized to receive co-design support with our partner, Onda Collective. Co-design is human-centered design approach that invites end-users into the design process as partners to improve acceptability, usability, and feasibility of intervention implementation. This approach will give site leads, clinicians, patients, and caregivers the opportunity to think through detailed aspects of how a specific intervention will be implemented together (Table 2). Sites will work with Onda’s trained designers to co-design one of their 2 to 3 interventions in their COPD TOC bundle.

**Table 2:** Features of mentored implementation and co-design approaches.

Feature	Mentored Implementation [44-50]	Co-design [51-55]
Goals	Culture change Practice change Capacity building Leadership development	Shared power Team alignment Integration of diverse expertise Meeting end-user needs and objectives
Approach	Consultative guidance Ongoing implementation support; expertise	Collaborative design Patients, staff, and clinicians as participants, not just “informants” Front-end process facilitation
Outputs	Monthly agendas, summary notes Virtual site visit records and reports Site implementation planning workbooks	Customer journey maps Design criteria for implementation and alignment Intervention implementation blueprints
Key activities	Mentorship meetings and virtual site visit	Participatory workshops
Team breakdown	Physicians with quality improvement expertise Project management support	Academically trained designers

### Randomization and Assignment of Interventions

Sites will be randomized to receive virtual or in-person delivery of COPD TOC programs along with either virtual or in-person MIM of these programs. Four study arms will thus be created, and sites will be randomized in a 1:1:1:1 fashion. Mentors will also be randomized to sites. Covariate-based constrained randomization will be used to achieve balance between groups. Mentor randomization will be performed via stratified randomization given uneven efforts of mentors; that is, some mentors worked at 2 sites while others did not.

### Outcomes

The primary effectiveness outcome for this study will be COPD-specific acute health care use, defined as a composite of all ED visits and hospitalizations within 30 days of index hospitalization for a COPD exacerbation. The primary implementation outcome will be reach, defined as the proportion of patients receiving their assigned TOC interventions (the whole bundle). Secondary effective outcomes will include additional acute care metrics related to COPD-specific and all-cause 30-, 60-, and 90-day revisits, ED visits, and readmissions. Secondary implementation outcomes include the reach of individual interventions within sites’ COPD TOC programs (ie, component-level reach).

### Data Collection

Quantitative data (aims 2 and 3): deidentified patient-level data related to program effectiveness and intervention delivery will be collected monthly on all patients discharged with an index hospitalization for COPD exacerbation and all subsequent encounters (Textbox 1). These data include patient demographics (eg, age, race, ethnicity, and gender), zip code (first 3 digits), smoking history, insurance, acute care use using specific *International Classification of Diseases, Tenth Revision* (*ICD-10*) codes, and all subsequent hospitalizations, ED visits, and relevant ambulatory visits (eg, primary care, pulmonology, and pulmonary rehabilitation). [56] The *ICD-10* codes used to identify COPD will be J40, J41.0, J41.1, J41.8, J42, J43.0, J43.1, J43.2, J43.8, J43.9, J44.0, J44.1, and J44.9. The *ICD-10* codes used to identify COPD-related hospitalizations will be J96.00, J96.01, J96.02, J96.10, J96.20, J96.21, J96.22, J96.90, and R09.2 WITH a secondary code for COPD. The interventions received (ie, patient education and post-discharge encounters) will also be captured.

Qualitative data (study aims 1 and 3): For both the contextual assessments (aim 1) and the sustainment assessments (aim 3), site leads will be first surveyed and then interviewed, followed by interviews with clinicians, organizational leaders, patients, and caregivers. For the contextual assessment, a final interview session with site leads was conducted to develop their site-specific implementation plans (manuscript in press). For the sustainment evaluations, we will collect data on sustainment, fidelity to the site implementation plan, and cost analysis (Tables 3 and 4).

**Deidentified patient-level data.**

**Identification**

- Fake medical record number (dummy number to track patients over time without being able to identify them)
- Fake encounter number (dummy number to track encounter-related data without being able to identify specific encounter)

**Encounter information**

- Encounter type
- Days since index discharge (we could not use real dates, sites provided us with the number of days since discharge from the index hospitalization for chronic obstructive pulmonary disease (COPD) whereby admission for the index was a negative number [0-day prior to discharge] and all subsequent encounters including ED visits, rehospitalizations, and outpatient visits were a positive number [0 + days until that encounter])
- Length of stay
- Discharge location

**Diagnosis codes (for the index COPD hospitalization, specific *International Classification of Diseases, Tenth Revision (ICD-10)* codes were used; for all other encounters we captured all *ICD-10* codes)**

- Primary *ICD-10* code
- Secondary *ICD-10* codes
- Diagnostic-related groups (optional)

**Characteristics**

- Primary insurance
- Secondary insurance
- Age at encounter
- Gender
- Race
- Ethnicity
- First 3 digits of zip code

**COPD data (optional)**

- Smoking status
- Disease duration
- Disease severity
- Pulmonary rehabilitation referral
- Smoking cessation referral
- Outpatient oxygen order
- Home health ordered

**Intervention data (tailored to each site whereby primary metric was reach (yes or no) with other metrics decided at the site level)**

- Interventions 1, 2, and 3 completed
- Other metrics for interventions 1, 2, and 3

**Table 3:** Sustainment, fidelity, and cost data collection by end-user group.

End-user group	Sustainment	Fidelity	Cost analysis
Site leads	✓	✓	✓
Organizational leaders	✓		
Clinicians	✓	✓	
Patients and caregivers	✓		

**Table 4:** Sustainment, fidelity, and cost domains.

Domain	Focus	End-user group	Data collection methods
Sustainment	Extent to which transitions of care (TOC) interventions are maintained post-implementation phase	Site leads, clinicians, organizational leaders, patients, caregivers	Survey, qualitative interviews
Fidelity	Degree to which sites adhere to their site-specific implementation plans	Site leads, clinicians	Survey, qualitative interviews
Cost analysis	Resources required to maintain the intervention and implementation supports	Site leads	Survey, qualitative interviews

### Statistical Analysis and Power Calculation

This primary analysis will be an intention-to-treat analysis with both unadjusted and adjusted analyses using generalized estimating equations. The primary intention-to-treat analysis will be applied to all enrolled hospitals and their outcomes, regardless of duration of intervention or implementation and actual adherence to the randomization at each site. Two-factor generalized estimating equations with a Poisson or negative distribution will be used to assess the effect of delivery mode (virtual vs in-person) and implementation support strategy (mentoring vs mentoring plus co-design), along with their interaction. Randomization “group 1” (deliver interventions virtually with mentoring plus co-design implementation support) will be compared to the remainder of the groups (Table 1). Covariates will include patient age, gender, and race, among others; hospital region; hospital type (academic vs community); setting (urban vs suburban vs rural); and hospital size. Models will also adjust for the baseline level of the primary outcomes at each individual hospital. Hospitals will be considered as a random intercept in a generalized linear mixed model, while within-hospital associations will be adjusted as clusters in a generalized estimating equation. We will also conduct pairwise comparisons among the four groups. For the primary effectiveness (30-day COPD-specific revisits) and the primary implementation outcome (reach of bundle), we will use 2-factor GEE with binomial distribution. A 2-sided type I error of 1% will be used for primary outcomes to account for multiple testing. Secondary outcomes, including other COPD-specific and all-cause 30-, 60-, and 90-day ED visits, readmissions, and revisits, will use the same methods as described above. These analyses will be exploratory and will not spend type I error of 5%. Time-to-event outcomes will be assessed via survival analysis with Cox proportional hazard regression models and Kaplan-Meier curves with the log-rank test. Tests of interaction to assess for any relationship between the intervention delivery (virtual versus in-person) and implementation support (mentoring only versus mentoring plus co-design) will also be performed. Given the testing of multiple hypotheses, a 2-sided *P* value of 1% will be considered significant. For analyses of secondary outcomes, a *P* value <5% will be considered significant regardless of type I error spending. For binary or count outcomes such as intervention provided (yes or no), the same statistical methods used in the primary analyses will be applied.

The sample size required for the trial is a minimum of 16 sites. For the primary effectiveness outcome of 30-day COPD-specific acute health care use, assuming a 30-day acute care revisit difference of 0.24 between virtual and in-person groups and a cluster size of 602, as was seen in pilot data [19,22,57], along with an intraclass correlation of 0.03, a total of 16 clusters and 4628 patients is needed to have 91% power to detect this difference. For the implementation outcomes of bundle reach, using a modified *z* test and assuming a 20% difference between receipt of interventions between arms and 90% power to detect a difference, 15 clusters with 4560 patients are needed. Both of these power calculations are at a 2-sided significance level of 1%.

### Sensitivity Analyses

We will assess the robustness of our findings with per-protocol and as-treated sensitivity analyses. Per protocol analysis is defined as comparing sites that adhered to their randomization for delivering interventions (all in person or all virtual), and as treated is defined as comparing sites regardless of randomization that delivered interventions all in person versus any virtual (hybrid virtual and in person). These will be repeated in each of the 4 arms, including testing for interaction between intervention strategy and implementation approach and among patient subgroups (eg, gender, age, and socioeconomic status) and hospital subgroups (region and hospital size). We will use 2-way generalized linear mixed models with hospitals considered as random intercepts.

## Results

As of August 2025, 21 sites completed the contextual assessments and developed site-specific implementation plans. Publication of the qualitative data from this pre-implementation phase is anticipated in December 2025. Randomization of sites has been performed, and the sites that were randomized to co-design have completed 3 sessions. Baseline use data collection is complete. Use data collection for the implementation year is nearly complete. Use data collection for years 1 and 2 of the post-implementation phase is ongoing. Quantitative data analyses of the baseline and implementation phase reports are nearly complete, with anticipation of submission of the primary implementation phase manuscript in December 2025, pending submission of final implementation-phase data reports. Anticipate manuscript submission for the post-implementation sustainment analyses is anticipated for September 2026. Qualitative data collection for year 1 of the post-implementation phase is complete, and analysis is under way. Qualitative data collection for year 2 is being initiated as of August 2025.

## Discussion

### Principal Findings

The Reduce REVISITS study will use novel integrated implementation science and human-centered design methodology to investigate bundles of effective COPD TOC interventions with the goal of reducing COPD hospital revisits. Using a virtual adaptation of the MIM strategy with or without co-design support, the interventions under study, delivered in person or virtually, will be tested for feasibility and endurance in addition to effectiveness. By specifically studying virtual delivery of these interventions and their implementation, our study evaluates whether this approach improves reach by integrating into the rapidly advancing telehealth landscape and may be optimally translated into practice even when resources are finite. Our study outcomes include both effectiveness and implementation measures to enhance dissemination and broader-scale implementation of COPD TOC programs.

Prior literature has investigated the effectiveness of multilevel interventions in a variety of disease states. Studies targeting the transition from the adolescent to young adult health systems, for instance, are ongoing and are using multimodal approaches to target information gaps among patients, health care providers, and caregivers [58]. Congestive heart failure and COPD readmission care bundles and post-discharge follow-up programs have been evaluated as a group for overall effectiveness with varying results, particularly for the outcomes of readmission and mortality [24,59,60]. However, which elements of these programs are the most effective and can be easily delivered to multiple health systems are current gaps in knowledge that the Reduce REVISITS study will fill.

The post-pandemic era has ushered in a plethora of virtual health care measures aimed at improving quality and decreasing cost [61]. Even before the pandemic, a randomized controlled trial found that a telehealth-based transitions of care program involving patients with multiple chronic diseases increased medication reconciliation and adherence compared to an in-person–based program, although telehealth did not reduce health care use in the form of ED visits or readmissions [62]. Specific to COPD, virtual pulmonary rehabilitation has been found to be comparably effective to in-person therapy, but routine clinical monitoring provided virtually has not been demonstrated to reduce health care use or cost compared to standard of care in-person visits [63,64]. The use of emerging wearable technologies for remote patient monitoring is also being actively investigated in relation to clinical outcomes in patients with congestive heart failure or COPD [65]. Ensuring the effectiveness of these virtual interventions through studies such as the Reduce REVISITS study and others will undoubtedly improve the care of chronically ill hospitalized patients.

While this study aims to find the most effective and scalable approaches to facilitate COPD TOC, it is unlikely that a one-size-fits-all approach exists for this complex, diverse, and comorbid patient population. Thus, we have specifically designed our study across a wide variety of hospital settings to capture as much of this variation as possible. Another potential limitation could be that patients could seek care outside of their previously used health care system, limiting data collection. By randomizing sites, we hope to mitigate this bias. We additionally will evaluate sites via value-based purchasing programs that have complete revisit data when possible.

In summary, TOC programs serve a vital role in the necessary goal of reducing admissions for COPD and other chronic diseases. The Reduce REVISITS study, with its novel methodology, wide scope of tested interventions, and introduction of co-design support, is poised to transform the landscape of multilevel systems-based interventions for this vulnerable patient population.

### Conclusions

The Reduce REVISITS study uses a pragmatic hybrid type II effectiveness-implementation design to evaluate COPD care transition programs delivered across hospital settings. By integrating mentored implementation with human-centered co-design, this work seeks to identify practical and scalable strategies for delivering evidence-based COPD TOC interventions in both virtual and in-person formats. The study will generate needed evidence on how implementation supports influence reach, effectiveness, and long-term sustainment. Findings will help guide hospitals in optimizing COPD care transitions and reducing preventable acute care use in this high-risk patient population.

## Appendix

Multimedia Appendix 1 Peer review report from DIRH - Dissemination and Implementation Research in Health Study Section, Healthcare Delivery and Methodologies Integrated Review Group (National Institutes of Health, USA).

## Abbreviations

ATS: American Thoracic Society
COPD: chronic obstructive pulmonary disease
ED: emergency department
HOMERuN: Hospital Medicine Reengineering Network
HRRP: Hospital Readmission Reduction Program
ICD-10: International Classification of Diseases, Tenth Revision
MIM: mentored implementation model
RCT: randomized controlled trial
REDCap: Research Electronic Data Capture
REVISITS: Respiratory Emergent Visits using Implementation Science Interventions Tailored to Setting
SHM: Society of Hospital Medicine
TOC: transition of care

## Data Availability

No data have been generated or analyzed yet, as this manuscript reports the study protocol. In accordance with the study’s registration on ClinicalTrials.gov (NCT05568043), individual participant data will not be shared. Upon study completion, aggregated results and related study materials (eg, survey instruments, codebooks, and analysis plans) will be made available in subsequent publications and, where appropriate, in National Institutes of Health–approved repositories in accordance with National Heart, Lung, and Blood Institute data-sharing policies and upon reasonable request.
