# Supplementary material for: Virtual Mentored Implementation to Improve Care Transitions in Chronic Obstructive Pulmonary Disease: Protocol for a Pragmatic Implementation Study
Source: JMIR Res Protoc. 2026 Jan 13;15:e82043. doi: 10.2196/82043 (PMC12848486; doi:10.2196/82043)
Supplement: Multimedia Appendix 1 [file resprot_v15i1e82043_app1.pdf]

PROGRAM CONTACT:
ANTONELLO PUNTURIERI
301-435-0230
punturieria@nhlbi.nih.gov

SUMMARY STATEMENT
(Privileged Communication)

Release Date: **11/18/2019**
Revised Date:

Application Number: **1 R01 HL146644-01A1**

Principal Investigator

PRESS, VALERIE G

Applicant Organization: UNIVERSITY OF CHICAGO

Review Group: **DIRH**
Dissemination and Implementation Research in Health Study Section

Meeting Date: **10/23/2019**
Council: **JAN 2020**
Requested Start: **04/01/2020**
RFA/PA: **PAR19-274**
PCC: **LLAA N**

Project Title: **The Virtual Mentored Implementation to Reduce REVISITS (Reducing Respiratory Emergent Visits using Implementation Science Interventions Tailored to Setting) Study**
SRG Action: **Impact Score:33 Percentile:25**
Next Steps: **Visit https://grants.nih.gov/grants/next_steps.htm**
Human Subjects: 30-Human subjects involved - Certified, no SRG concerns
Animal Subjects: 10-No live vertebrate animals involved for competing appl.
Gender: 1A-Both genders, scientifically acceptable
Minority: 1A-Minorities and non-minorities, scientifically acceptable
Age: 3A-No children included, scientifically acceptable

Project Year	Direct Costs Requested	Estimated Total Cost
1	499,788	799,752
2	499,986	800,069
3	499,859	799,866
4	499,998	800,088
5	499,842	799,839
TOTAL	2,499,473	3,999,615

ADMINISTRATIVE BUDGET NOTE: The budget shown is the requested budget and has not been adjusted to reflect any recommendations made by reviewers. If an award is planned, the costs will be calculated by Institute grants management staff based on the recommendations outlined below in the COMMITTEE BUDGET RECOMMENDATIONS section.
EARLY STAGE INVESTIGATOR
NEW INVESTIGATOR

1R01HL146644-01A1 Press, Valerie

EARLY STAGE INVESTIGATOR
NEW INVESTIGATOR

RESUME AND SUMMARY OF DISCUSSION: This R01 application proposes to use implementation science methods to compare the effectiveness of a virtual versus in-person multi-level Chronic Obstructive Pulmonary Disease (COPD) care transition program in order to determine which implementation increases the program's reach. The scientific premise for the proposed work is strengthened by including preliminary evidence that supports the effectiveness of each intervention, including recent findings from this group. This is a strong team of investigators, led by a highly-skilled new investigator. The multidisciplinary team includes expertise in appropriate disciplines, such as COPD, implementation science, and health economics; reviewers agree that this team has the expertise and experience to complete the proposed project. The planned comparison is innovative; no prior study has compared a virtual multi-level program with a mentored implementation approach. The proposed multi-level COPD care transition program incorporates key interventions already found to be individually effective. The approach is robust in term of the number and diversity of sites, with good representation of rural, urban, and suburban patient populations. However, coordination of this study may present a challenge, given its complexity, with there being some concern about feasibility. This resubmission is responsive to concerns raised in the initial review, with the study design being significantly modified. For example, the intervention is now multi-stage. Procedures have been revised so that patients will regularly receive interventions during and after hospitalization. The proposed statistics plan is appropriate and has been improved in this resubmission by expanding proposed cost analyses. However, the results may not provide strong evidence of the effectiveness of virtual education, since the distinction between delivery types is nebulous. Consequently, this revision is a missed opportunity to assess drivers of implementation. Finally, concern remains that the proposed intervention will not account for the complexity of care, or capture factors, besides care transition programs, that may affect patient outcomes. Overall, the review panel agrees that the premise for the proposed research has been strengthened in this resubmission by revising the methodology and including supporting preliminary data. However, despite revisions to the approach, the overall impact is reduced to moderate to high by methodological shortcomings, including study arms that are insufficiently distinct for clear assessments of delivery formats.

DESCRIPTION (provided by applicant): Chronic Obstructive Pulmonary Disease (COPD) affects 16 million US adults, many of whom experience high rates of emergency department and hospital COPD revisits after initial hospitalizations due to care transition failures. These frequent COPD exacerbations lead to more rapid lung function decline and earlier mortality. Further, hospitalizations for exacerbations highly contribute to the ~$50 billion spent annually for COPD care in the US. Therefore, COPD revisits are now a public health crisis. It is feasible to improve COPD care and decrease acute care revisits, as shown by published evidence of successful care transition interventions. Our team has led efforts to identify effective care transition interventions and has successfully piloted a multi-level COPD care transition program. Effective care transition interventions include medication reconciliation, self-management education, and post-discharge communication. However, for wide-spread adoption to occur, we must identify optimal intervention delivery methods based on hospitals' resources and patient care needs. For instance, virtually-supported interventions are often more resource-friendly, and while effectiveness data on individual virtual interventions exists, multi-level virtual programs have not yet been studied compared to in- person programs. In addition, feasible implementation approaches to support the delivery of evidence-based care transition programs are needed for wide-scaled dissemination and sustainability. Our team has found that a mentored implementation model is effective for implementing multi-level, hospital-based programs across US health systems. This approach traditionally relies on in-person site visits. The use of virtual site visits could dramatically increase this model's reach, but has not yet been studied. In summary, for successful, wide-scale adoption, diverse US hospitals need to have access to feasible, multi-level care transition programs and effective

implementation approaches that are aligned with site-specific care needs and resources, but currently the optimal approach is unknown. Thus, in this proposal, we will compare the effectiveness of virtual vs. in- person multi-level COPD care transition programs in real-world settings by concurrently studying whether virtual or in-person mentored implementation increases programs' reach. We will collaborate with the Hospital Medicine Reengineering Network convened for rapid discovery and dissemination to identify and enroll sites. After conducting pre-implementation contextual assessments at all sites using the Consolidated Framework for Implementation Research, we will conduct a Hybrid Type II Effectiveness-Implementation study to determine effectiveness of the programs to reduce 30-day COPD revisits and of the mentored implementation to increase program penetration. Finally, we will study programs' sustained outcomes for two years post-implementation. Data from this study will inform the optimal implementation of COPD care transition programs at scale. Further, lessons gleaned from this study can inform implementation approaches for other hospital-based programs.

PUBLIC HEALTH RELEVANCE: Chronic Obstructive Pulmonary Disease (COPD) burdens over 16 million US adults and frequently results in recurrent emergency department visits and hospitalizations due to poor care transition support after initial COPD exacerbations. Our study will use innovative and rigorous implementation science methods to identify real-world solutions to decreasing COPD acute care revisits by concurrently studying care transition program intervention delivery methods (virtual versus in-person) and mentored implementation approaches (virtual or in-person). Lessons learned from this study could transform the way in which care is delivered to millions of COPD patients across the US and could even be applied widely to improve the effectiveness and sustainability of other multi-level, hospital-based programs.

CRITIQUE 1

Significance: 3
Investigator(s): 1
Innovation: 3
Approach: 3
Environment: 1

Overall Impact: This is an R01 resubmission of a proposal submitted by an early-stage and new investigator to: conduct a Hybrid Type II Effectiveness-Implementation study to determine the effectiveness (Aim 2) of a 4-arm trial, program delivery/mentored implementation dyads: virtual vs in-person multi-level COPD care transition program to decrease COPD readmission rates, AND virtual vs in-person mentored implementation support to increase program penetrance. Aim 1 for this study builds upon strong implementation frameworks to assess the pre-implementation context, program implementation (Aim 1), and post-implementation sustainability (Aim 3) of the COPD program. This is a strong team of collaborators who have demonstrated their ability to conduct large-scale studies in their previous work. Expertise in COPD, implementation science, and all other relevant fields (like biostatistics and health economics) rounds out the team's ability to successfully conduct this research. The team has modified the intervention to be multi-staged and has some preliminary evidence to support the scientific premise. However, the premise is weakened by the lack of control over what is actually being implemented, and therefore the true distinction between the study arms for Aim 2 is blurred. The methodological approach to achieve each aim is moderate to strong.

1. Significance:
Strengths

- The scientific premise for the multi-level care program to prevent COPD readmissions is moderate to strong and is primarily based on numerous studies of the individual components of the program; and recently published results on a multi-level program introduced at the University of Chicago, where readmission rates were reduced by 30% in 1 ½ years with a multi-

level care program. Even though there is limited evidence of the effectiveness of a multi-level program, guidelines for the field are promoting these programs and their evaluation.
- The scientific premise for including the Mentored Implementation Model (MIM), which pairs clinical national experts with implementing sites, as another intervention component under consideration is strong and based on two large studies (MARQUIS and BOOST) led by the Society of Hospital Medicine (SHM), co-investigators on this study.
- There is considerable evidence from the field that virtual mentoring is effective, including from the study team (ECHO for asthma); and for patient management.
- Strong theoretical models in implementation science, CFIR and Proctor's model, will guide intervention development/site-level adaptability (Aim 1) and sustainability (Aim 3)

Weaknesses
- There is less evidence that virtual patient education is effective, although the team has one paper under review that demonstrates non-inferiority between virtual vs in-person Teach to Go (TTG), rounds of demonstrations and re-assessments for patient training on proper inhaler usage.

2. Investigator(s):
Strengths
- Dr. Press is a physician who has established herself as an expert in hospital-based COPD care, studying factors that lead to 30-day COPD revisits. She is currently leading her institution's effort to reduce acute care visits for asthma and COPD among patients in primary care practice and patients who present to the emergency department. Her expertise has led to appointments in the American Thoracic Society, Society of Hospital Medicine COPD expert panel, the opportunity to review the clinical guidelines for screening for COPD published in *JAMA*, *Lancet Respiratory Medicine* review of COPD care, and she was named as the Executive Medical Director of Value Based Care for the Center for Care Transformation.
- Dr. Erwin, co-I, recently led the Contextual Inquiry stage of a study to develop a locally tailored, multi-level asthma intervention asthma care implementation plan using the RE-AIM framework. She is also a leader in Human Centered Design (HCD) methods. Dr. Damschroder is the developer of the CFIR framework. This mirrors the plans for Aim 1 in this study.
- The team also includes clinical content expertise (White), health economics (Konetzka), statistics (Wan), and the CFIR and Procter frameworks (Damschroder, consultant), and Mentored Implementation Model (MIM) programming (Goldstein).

Weaknesses
- Coordination of site leads may be challenging. There are 2 two site lead members at each of the 20 sites (for Aim 1 in year 1), n=16 sites (for Aim 2 in years 2-4), and n=8 sites (for Aim 3 in year 5).

3. Innovation:
Strengths
- For Aim 1, the multi-site (n=20) assessment of baseline care transition interventions and their variation in delivery within and across hospitals will obtain novel data.
- This innovative study design will allow the team to identify effective and sustainable solutions to gaps in delivering evidence-based COPD care.

Weaknesses
- There is a missed opportunity to collect person-level data to determine patient characteristics that may be associated with implementation effectiveness.

4. Approach:
Strengths
- All sites (n=20 selected from a list of 60 hospitals in a program for rapid dissemination; n=26 letters of support included in proposal) will participate in a pre-implementation context assessment [Aim 1] using the Consolidated Framework for Implementation Science (CFIR) and

human-centered design (HCD; e.g., direct observation) to identify care variation and implementation plans. The goal is to determine site-specific interventions, resources, team members, and data collection and reporting systems, and to refine multi-level programming for Aim 2.

- For Aim 1, data will be collected from patients and providers (n=5/hospital), and key administrators (n=2-3) using semi-structured interviews based on all 5 domains of the CFIR. Purposeful sampling will be conducted to account for demographic differences (patients), and professional differences (clinicians). Semi-structured interviews will be conducted, (using appropriate qualitative methods of transcribing, coding and analyzing) and including more sample if saturation isn't achieved.
- For Aim 2, at least 16 sites will be randomized to one of the 4 arms of the effectiveness trial, to evaluate the comparative effectiveness of program delivery method (virtual vs in-person) to decrease COPD revisits (in the aggregate, at the hospital level) and the mentored implementation approach (virtual vs in-person) to increase program penetration. The statistical analysis is rigorous: a two-factor generalized estimating equations (GEE) will be conducted to account for within-site associations and a multilevel model, i.e., a two-factor generalized linear mixed model (GLMM) will be conducted. Aggregate-level patient- and hospital-level covariates including patients' basic demographic characteristics (age, gender, and race), state, geographical location of hospitals (urban vs. rural), and hospital size will be incorporated. Hospital-level baseline outcomes (e.g., number of acute care events in prior year), will be adjusted in the models. A two-sided p-value <1% will be considered significant. Additional one-factor analysis will be considered.
- In Aim 3 (post-implementation), the sustainability of intervention and implementation outcomes will be evaluated at 6, 12, 18, and 24-months post-implementation. Mechanisms, moderators, and mediators will identify aspects of successful implementation, and program costs and savings will be analyzed across sites.
- The cost analysis is conducted at the provider (hospital) level, and includes costs/savings related to implementation at the hospital (start-up, personnel, space/materials) and also costs at the overall intervention level (training, mentoring, systems to collect data etc.).

Weaknesses

- There is some concern that what is actually being delivered in the multi-level COPD program will not be similar enough across the arms to be comparable.
- There also may be overlap in the delivery format of in-person and virtual (some components could be in-person, some could be virtual).
- Providers are not blinded to the study hypotheses.
- Data on effectiveness are collected at the aggregate-level, not individual, thus preventing any meaningful understanding of patient-level characteristics that may be associated with implementation success. Further, cost-effectiveness analyses are not viable with data collected in the aggregate.

5. Environment:

Strengths

- University of Chicago and the relevant research centers and institutes provide a strong infrastructure to support collaborate research.
- Multiple sites for co-investigators include: COPD Foundation, Baystate Medical Center, The Society of Hospital Medicine, University of Illinois.
- The University of Chicago Medicine will be the IRB of record for all components of this study.

Weaknesses

- None noted by reviewer

Study Timeline:

Strengths

- Acceptable.

Weaknesses

- None noted by reviewer

Protections for Human Subjects:
Acceptable Risks and/or Adequate Protections

- The risks to humans are minimal. Informed consent will be obtained for those participating in semi-structured interviews for Aims 1 and 3 only. But they will obtain waivers of consent for each hospital site participating in the study to collect hospital-level and de-identified patient-level data.

Data and Safety Monitoring Plan (Applicable for Clinical Trials Only):
Acceptable

- A DSMB will be assigned, even though the risks to humans is minimal. This DSMB will consist of 8 members across multiple institutions with broad and deep expertise, who will be involved with the safety and monitoring of this study. All members of the DSMB are independent individuals that are not otherwise involved with the research and have research expertise in patient experience, statistical methods, recruitment and retention, and intervention studies.

Inclusion of Women, Minorities and Children:

- Sex/Gender: Distribution justified scientifically
- Race/Ethnicity: Distribution justified scientifically
- For NIH-Defined Phase III trials, Plans for valid design and analysis: Not applicable
- Inclusion/Exclusion of Children under 18: Excluding ages <18; justified scientifically
- Stakeholder data collected evenly across males/females; this is an adult disease.

Vertebrate Animals:
Not Applicable (No Vertebrate Animals)

Biohazards:
Not Applicable (No Biohazards)

Resubmission:

- The cover page states that modifications to the proposal are denoted with red bars, yet these bars are not visible. This makes it very difficult to see where actual content has been modified to address reviewer concerns. The main changes include: a multi-stage COPD intervention (not just patient education), preliminary data from a multi-stage intervention implemented at UChicago (not yet published). The cost analysis now also includes some assessment of possible savings resulting from reduced readmissions.

Applications from Foreign Organizations:
Not Applicable (No Foreign Organizations)

Select Agents:
Not Applicable (No Select Agents)

Resource Sharing Plans:
Acceptable

Authentication of Key Biological and/or Chemical Resources:
Not Applicable (No Relevant Resources)

Budget and Period of Support:
Recommend as Requested

CRITIQUE 2

Significance: 4
Investigator(s): 2
Innovation: 5
Approach: 7
Environment: 2

Overall Impact: The investigators will compare the effectiveness of virtual vs. in person multi-level COPD care transition programs in real-world settings by concurrently studying whether virtual or in-person mentored implementation increases programs' reach by collaborating with the Hospital Medicine Reengineering Network convened for rapid discovery and dissemination to identify and enroll sites. There are several issues with the proposed research that will greatly limit its impact including a rather simplistic intervention which has not been tested in a pilot study for feasibility and fidelity. The evaluation of the effectiveness of the intervention is also not well designed. How this intervention differs significantly from currently used approaches and what are their current limitations need to be thoroughly examined. Overall the virtual care transition programs are not particularly innovative in chronic disease patient management and the proposed research will not further our understanding of implementation of programs to improve patient outcomes in COPD significantly as proposed.

1. Significance:
Strengths
- The investigators propose to use innovative and rigorous implementation science methods to identify real-world solutions to decreasing COPD acute care revisits by concurrently studying care transition program intervention delivery methods (virtual versus in-person) and mentored implementation approaches (virtual or in-person)

Weaknesses
- There are many other extraneous factors besides the care transition program that affect patient outcomes that are not captured by the current study design.

2. Investigator(s):
Strengths
- The investigators are well qualified to conduct the research. The PI is a junior clinician researcher. The study team contains many strong researchers

Weaknesses
- None noted by reviewer

3. Innovation:
Strengths
- None noted by reviewer

Weaknesses
- The use of virtual patient mentoring in chronic disease patient care is not particularly novel although this may be the first time it is formally evaluated in a large group of COPD patients.

4. Approach:
Strengths
- None noted by reviewer

Weaknesses
- There are many factors besides the care management program that could affect patient outcomes and the current study design not capture them.

- The rather simplistic intervention is unlikely to account for the complexity of care of the multimorbid COPD patient and has not been tested in pilot studies for fidelity and feasibility.
- The modeling of patient outcomes is not well described and how patient selection and confounding by indication will be addressed is not clear.
- The investigators have not presented a thorough evaluation of current COPD patient management approaches and how their virtually delivered mentoring intervention will overcome current clinical practice limitations.

5. Environment:
Strengths
- The environment and resources at the University of Chicago are excellent for conduct of this research.

Weaknesses
- None noted by reviewer

Study Timeline:
Strengths
- Detailed timeline is presented.

Weaknesses
- None noted by reviewer

Protections for Human Subjects:
Acceptable Risks and/or Adequate Protections
- This is described in a separate section

Data and Safety Monitoring Plan (Applicable for Clinical Trials Only):

Inclusion of Women, Minorities and Children:
- Sex/Gender: Distribution justified scientifically
- Race/Ethnicity: Distribution justified scientifically
- For NIH-Defined Phase III trials, Plans for valid design and analysis:
- Inclusion/Exclusion of Children under 18:

Vertebrate Animals:
Not Applicable (No Vertebrate Animals)

Biohazards:
Not Applicable (No Biohazards)

Applications from Foreign Organizations:
Not Applicable (No Foreign Organizations)

Select Agents:
Not Applicable (No Select Agents)

Resource Sharing Plans:
Not Applicable (No Relevant Resources)

Authentication of Key Biological and/or Chemical Resources:
Not Applicable (No Relevant Resources)

Budget and Period of Support:
Recommend as Requested

CRITIQUE 3

Significance: 3
Investigator(s): 2
Innovation: 3
Approach: 3
Environment: 1

Overall Impact: The proposal aims to conduct a hybrid Type II Effectiveness-Implementation study of multi-level COPD care transition programs to assess whether virtual or in-person mentored implementation increases the program's reach. The proposed study is feasible to decrease acute care COPD revisits. The research team has developed a Mentored Implementation Model (MIM). The MIM pairs local clinical champions with national experts and has led to successful multi-level program implementation. The MIM traditionally relies on in-person site visits; however, virtual visits may increase MIM's scalability. A minor issue could be a lack of pilot study showing the strong effect sizes of virtual implementation as compared to mentored implementation, though the research team did conduct a pilot study with a large sample size to demonstrate the feasibility of the implementation.

1. Significance:
Strengths
- Virtual visits may increase the MIM pairs' scalability.

Weaknesses
- A minor issue could be a lack of pilot study showing the strong effect sizes of virtual implementation as compared to mentored implementation, though the research team did conduct a pilot study with a large sample size to demonstrate the feasibility of the implementation.

2. Investigator(s):
Strengths
- PI has accumulated rich experience in leading and completing multiple small grants.

Weaknesses
- None noted by reviewer

3. Innovation:
Strengths
- Testing both a virtual multi-level program and mentored implementation approach is novel.

Weaknesses
- None noted by reviewer

4. Approach:
Strengths
- The research team proposes a multi-level COPD care transition program that incorporates key interventions already found to be individually effective, such as medication reconciliation, self-management education, and post-discharge communication. (major)
- The revised proposal will ensure that COPD patients regularly and consistently receive interventions, not just once but on multiple occasions during and after hospitalization. (major)
- 24 clinical sites are from diverse US geographic regions representing rural, urban, and suburban patient populations. (major)
- The statistical analysis plan is appropriate.

Weaknesses
- The complete cost-effective analysis is not provided, although the research did expand the cost analysis to include program costs, implementation costs, and estimated hospital-related savings

to better estimate the value of implementing the COPD program interventions across the diverse hospital sites. (minor)

5. Environment:
Strengths
- The research environment is excellent and is suited to complete the proposed study.

Weaknesses
- None noted by reviewer

Study Timeline:
Strengths
- The timeline appears appropriate to allow for completion of the proposed study.

Weaknesses
- None noted by reviewer

Protections for Human Subjects:
Acceptable Risks and/or Adequate Protections
- Details of protection of human subjects are provided

Data and Safety Monitoring Plan (Applicable for Clinical Trials Only):
Acceptable
- Details of DSM are provided.

Inclusion of Women, Minorities and Children:
- Sex/Gender: Distribution justified scientifically
- Race/Ethnicity: Distribution justified scientifically
- For NIH-Defined Phase III trials, Plans for valid design and analysis: Not applicable
- Inclusion/Exclusion of Children under 18: Excluding ages <18; justified scientifically
- Youth with ages < 18 have a low prevalence of COPD.

Vertebrate Animals:
Not Applicable (No Vertebrate Animals)

Biohazards:
Not Applicable (No Biohazards)

Resubmission:
- The application is responsive to the previous critiques.

Applications from Foreign Organizations:
Not Applicable (No Foreign Organizations)

Select Agents:
Not Applicable (No Select Agents)

Resource Sharing Plans:
Acceptable

Authentication of Key Biological and/or Chemical Resources:
Not Applicable (No Relevant Resources)

Budget and Period of Support:
Recommend as Requested

THE FOLLOWING SECTIONS WERE PREPARED BY THE SCIENTIFIC REVIEW OFFICER TO SUMMARIZE THE OUTCOME OF DISCUSSIONS OF THE REVIEW COMMITTEE, OR REVIEWERS' WRITTEN CRITIQUES, ON THE FOLLOWING ISSUES:

PROTECTION OF HUMAN SUBJECTS: ACCEPTABLE

INCLUSION OF WOMEN PLAN: ACCEPTABLE

INCLUSION OF MINORITIES PLAN: ACCEPTABLE

INCLUSION ACROSS THE LIFESPAN PLAN: ACCEPTABLE

COMMITTEE BUDGET RECOMMENDATIONS: The budget was recommended as requested.

Footnotes for 1 R01 HL146644-01A1; PI Name: Press, Valerie G

NIH has modified its policy regarding the receipt of resubmissions (amended applications). See Guide Notice NOT-OD-14-074 at http://grants.nih.gov/grants/guide/notice-files/NOT-OD-14-074.html. The impact/priority score is calculated after discussion of an application by averaging the overall scores (1-9) given by all voting reviewers on the committee and multiplying by 10. The criterion scores are submitted prior to the meeting by the individual reviewers assigned to an application, and are not discussed specifically at the review meeting or calculated into the overall impact score. Some applications also receive a percentile ranking. For details on the review process, see http://grants.nih.gov/grants/peer_review_process.htm#scoring.

MEETING ROSTER

Dissemination and Implementation Research in Health Study Section
Healthcare Delivery and Methodologies Integrated Review Group
CENTER FOR SCIENTIFIC REVIEW
DIRH
10/23/2019 - 10/24/2019

Notice of NIH Policy to All Applicants: Meeting rosters are provided for information purposes only. Applicant investigators and institutional officials must not communicate directly with study section members about an application before or after the review. Failure to observe this policy will create a serious breach of integrity in the peer review process, and may lead to actions outlined in NOT-OD-14-073 at https://grants.nih.gov/grants/guide/notice-files/NOT-OD-14-073.html and NOT-OD-15-106 at https://grants.nih.gov/grants/guide/notice-files/NOT-OD-15-106.html, including removal of the application from immediate review.

CHAIRPERSON(S)

BARTELS, STEPHEN J, MD, MS
DIRECTOR, MONGAN INSTITUTE
PROFESSOR OF MEDICINE
MASSACHUSETTS GENERAL HOSPITAL
HARVARD UNIVERSITY
BOSTON, MA 02114

MEMBERS

AALSMA, MATTHEW, PHD
PROFESSOR
DEPARTMENT OF PEDIATRICS
SCHOOL OF MEDICINE
INDIANA UNIVERSITY
INDIANAPOLIS, IN 46203

ADEBAMOWO, CLEMENT ADEBAYO, MD
PROFESSOR
DEPARTMENT OF EPIDEMIOLOGY AND PUBLIC HEALTH
UNIVERSITY OF MARYLAND SCHOOL OF MEDICINE
BALTIMORE, MD 21201

ALHASSAN, SOFIYA, BS, MS, PHD *
ASSOCIATE PROFESSOR
DEPARTMENT OF KINESIOLOGY
UNIVERSITY OF MASSACHUSETTS AMHERST
AMHERST, MA 01003

ARCOLEO, KIMBERLY JOAN, PHD *
PRINCIPAL INVESTIGATOR AND PROFESSOR
RESEARCH INSTITUTE
AT NATIONWIDE CHILDREN'S HOSPITAL
PEDIATRICS MEDICAL CENTER
THE OHIO STATE UNIVERSITY
COLUMBUS, OH 14642

BALKRISHNAN, RAJESH, PHD *
PROFESSOR AND DIRECTOR
POPULATION HEALTH AND PREVENTION SCIENCES
CANCER CONTROL CORE AT THE UVA CANCER CENTER
SCHOOL OF MEDICINE AND SCHOOL OF NURSING
UNIVERSITY OF VIRGINIA
CHARLOTTESVILLE, VA 22908

BECAN, JENNIFER RENEA, PHD *
ASSOCIATE RESEARCH SCIENTIST
INSTITUTE OF BEHAVIORAL RESEARCH
TEXAS CHRISTIAN UNIVERSITY
FORT WORTH, TX 76129

BRANDT, HEATHER M, PHD
ASSOCIATE DEAN AND PROFESSOR
HEALTH PROMOTION, EDUCATION, AND BEHAVIOR
ARNOLD SCHOOL OF PUBLIC HEALTH
UNIVERSITY OF SOUTH CAROLINA
COLUMBIA, SC 29208

BROWN, JEREMIAH R, PHD *
ASSOCIATE PROFESSOR
HEALTH POLICY AND CLINICAL PRACTICE
GEISEL SCHOOL OF MEDICINE
THE DARTMOUTH INSTITUTE
LEBANON, NH 03756

CARRASQUILLO, OLVEEN, MD, MPH
PROFESSOR OF MEDICINE AND PUBLIC HEALTH SCIENCES
CHIEF, DIVISION OF INTERNAL MEDICINE GERIATRICS
MILLER SCHOOL OF MEDICINE
UNIVERSITY OF MIAMI
MIAMI, FL 33101

CHARLEBOIS, EDWIN DUNCAN, PHD *
PROFESSOR
DEPARTMENT OF MEDICINE
SCHOOL OF MEDICINE
UNIVERSITY OF CALFORNIA, SAN FRANCISCO
SAN FRANCISCO, CA 94105

CHI, BENJAMIN H, MD *
PROFESSOR
DEPARTMENT OF OBSTETRICS AND GYNECOLOGY
SCHOOL OF MEDICINE
UNIVERSITY OF NORTH CAROLINA AT CHAPEL HILL
CHAPEL HILL, NC 27514

CORSO, PHAEDRA S, PHD
VICE PRESIDENT FOR RESEARCH
OFFICE OF RESEARCH
KENNESAW STATE UNIVERSITY
KENNESAW, GA 30144

DAI, HONGYING, PHD *
ASSOCIATE PROFESSOR
DEPARTMENT OF BIOSTATISTICS
UNIVERSITY OF NEBRASKA
MEDICAL CENTER
OMAHA, NE 68198--437

EIRALDI, RICARDO B, PHD
ASSOCIATE PROFESSOR
DEPARTMENT OF PEDIATRICS AND PSYCHIATRY
CHILDREN'S HOSPITAL OF PHILADELPHIA
UNIVERSITY OF PENNSYLVANIA
PERELMAN SCHOOL OF MEDICINE
PHILADELPHIA, PA 19146

FERKETICH, AMY K, PHD *
PROFESSOR
DIVISION OF EPIDEMIOLOGY
COLLEGE OF PUBLIC HEALTH
OHIO STATE UNIVERSITY
COLUMBUS, OH 43210

GRIFFIN, BETH ANN, PHD
SENIOR STATISTICIAN
RAND CORPORATION
ARLINGTON, VA 22202

HANNON, MARGARET, MPH, PHD
PROFESSOR
DEPARTMENT OF HEALTH SERVICES
UNIVERSITY OF WASHINGTON
SEATTLE, WA 98105

HOWARD, DAVID H, BA, PHD *
PROFESSOR
DEPARTMENT OF HEALTH POLICY AND MANAGEMENT
ROLLINS SCHOOL OF PUBLIC HEALTH
EMORY UNIVERSITY
ATLANTA, GA 30322

JOHNSTON, FABIAN M, MD *
ASSOCIATE PROFESSOR
DIRECTOR, PERITONEAL SURFACE MALIGNANCY PROGRAM
DIVISION OF SURGICAL ONCOLOGY
JOHNS HOPKINS UNIVERSITY
BALTIMORE, MD 21287

KEAN, JACOB T, BS, MA, MED, PHD *
ASSOCIATE PROFESSOR
DEPARTMENT OF POPULATION HEALTH SCIENCES
UNIVERSITY OF UTAH SCHOOL OF MEDICINE
SALT LAKE CITY, UT 84108

KUSHEL, MARGOT B, MD
PROFESSOR AND DIRECTOR
UCSF CENTER FOR VULNERABLE POPULATIONS
DIVISION OF GENERAL INTERNAL MEDICINE
ZUCKERBERG SAN FRANCISCO GENERAL HOSPITAL
UNIVERSITY OF CALIFORNIA SAN FRANCISCO
SAN FRANCISCO, CA 94143

LELUTIU-WEINBERGER, CORINA, PHD *
ASSISTANT PROFESSOR AND ENDOWED CHAIR OF THE
FRANÇOIS-XAVIER BAGNOUD CENTER
RUTGERS BIOMEDICAL AND HEALTH SCIENCES
SCHOOL OF NURSING
RUTGERS UNIVERSITY
NEWARK, NJ 07101

LI, JING, MD, MS *
ASSOCIATE PROFESSOR AND CO-DIRECTOR
CENTER FOR HEALTH SERVICES RESEARCH (CHSR)
DEPARTMENT OF INTERNAL MEDICINE
UNIVERSITY OF KENTUCKY
DIRECTOR, OVIHD, UK HEALTHCARE
LEXINGTON, KY 40536-0284

MANN, DEVIN M, MD
ASSOCIATE PROFESSOR
DEPARTMENT OF POPULATION HEALTH
NEW YORK UNIVERSITY SCHOOL OF MEDICINE
NEW YORK, NY 10016

MCLEOD, BRYCE DOUGLAS, PHD
PROFESSOR
DEPARTMENT OF PSYCHOLOGY
VIRGINIA COMMONWEALTH UNIVERSITY
RICHMOND, VA 23284

PALINKAS, LAWRENCE A, PHD
PROFESSOR
SUZANNE DWORAK-PECK SCHOOL OF SOCIAL WORK
UNIVERSITY OF SOUTHERN CALIFORNIA
LOS ANGELES, CA 90089

PATIL, CRYSTAL LAUREN, PHD *
PROFESSOR AND DEPARTMENT HEAD
DEPARTMENT OF WOMEN, CHILDREN AND FAMILY
HEALTH SCIENCE
COLLEGE OF NURSING
UNIVERSITY OF ILLINOIS AT CHICAGO
CHICAGO, IL 60612

POLLINI, ROBIN A, PHD
ASSOCIATE PROFESSOR
DEPARTMENT OF BEHAVIORAL MEDICINE AND PSYCHIATRY
SCHOOL OF MEDICINE
WEST VIRGINIA UNIVERSITY
MORGANTOWN, WV 26506

REZNIK, MARINA, MD
PROFESSOR OF PEDIATRICS
DEPARTMENT OF PEDIATRICS
DIVISION OF ACADEMIC GENERAL PEDIATRICS
THE CHILDREN'S HOSPITAL AT MONTEFIORE
ALBERT EINSTEIN COLLEGE OF MEDICINE
BRONX, NY 10467

SARPONG, DANIEL F, PHD
PROFESSOR OF BIOSTATISTICS, ENDOWED CHAIR AND
DIRECTOR
CENTER FOR MINORITY HEALTH AND HEALTH DISPARITIES
RESEARCH AND EDUCATION
COLLEGE OF PHARMACY
XAVIER UNIVERSITY
NEW ORLEANS, LA 70125

SKOLARUS, TED ALBERT, MD, MPH
ASSOCIATE PROFESSOR AND RESEARCH SCIENTIST
VA ANN ARBOR HEALTHCARE SYSTEM
DEPARTMENT OF UROLOGY
UNIVERSITY OF MICHIGAN
ANN ARBOR, MI 48109

TANNER, AMANDA E, BA, MPH, PHD *
ASSOCIATE PROFESSOR
DEPARTMENT OF PUBLIC HEALTH EDUCATION
SCHOOL OF HEALTH AND HUMAN SCIENCES
UNIVERSITY OF NORTH CAROLINA GREENSBORO
GREENSBORO, NC 27402

WAINBERG, MILTON L, MD *
PROFESSOR OF CLINICAL PSYCHIATRY
DEPARTMENT OF PSYCHIATRY
COLLEGE OF PHYSICIANS AND SURGEONS
COLUMBIA UNIVERSITY
NEW YORK, NY 10032

WHEELER, DARRELL P, PHD *
PROVOST AND SENIOR VICE PRESIDENT FOR ACADEMIC AFFAIRS
IONA COLLEGE
NEW ROCHELLE, NY 10801

SCIENTIFIC REVIEW OFFICER

FOSU, GABRIEL B, PHD
SCIENTIFIC REVIEW OFFICER
CENTER FOR SCIENTIFIC REVIEW
NATIONAL INSTITUTES OF HEALTH
BETHESDA, MD 20892

EXTRAMURAL SUPPORT ASSISTANT

JONES, BELINDA
EXTRAMURAL SUPPORT ASSISTANT
CENTER FOR SCIENTIFIC REVIEW
NATIONAL INSTITUTES OF HEALTH
BETHESDA, MD 20892

* Temporary Member. For grant applications, temporary members may participate in the entire meeting or may review only selected applications as needed.

Consultants are required to absent themselves from the room during the review of any application if their presence would constitute or appear to constitute a conflict of interest.
